# Molecular Simulation-Guided Design of H-Bonding-Reinforced Trihydroxy-Phenolics/Poly(vinyl alcohol) Composite Hydrogel

**DOI:** 10.3390/molecules31152595

**Published:** 2026-07-24

**Authors:** Jie Chen, Ying Zhou, Yishao Wang, Fujing Huang, Fangming Zou, Shunmei He, Xianwen Song

**Affiliations:** 1 Guangxi Universities Engineering Research Center for Innovative Product Development of Regional Priority Diseases, Guangxi University of Chinese Medicine, Nanning 530001, China; chenj2024@gxtcmu.edu.cn; 2 Institute of Traditional Chinese and Zhuang-Yao Ethnic Medicine, Guangxi University of Chinese Medicine, Nanning 530001, China; 3 Engineering Research Center of Innovative Traditional Chinese, Zhuang and Yao Materia Medica, Ministry of Education, Nanning 530001, China; 4 School of Pharmacy, Guangxi University of Chinese Medicine, Nanning 530001, China; zhouying.zyy@foxmail.com (Y.Z.); wang_623@foxmail.com (Y.W.); huangfujingshuaige@foxmail.com (F.H.); fangmingzou.zfm@foxmail.com (F.Z.); 5 School of Chemistry and Material Science, Hunan Agricultural University, Changsha 410083, China

**Keywords:** PVA, phenolics, hydrogel, H-bonding, crosslinking

## Abstract

Small-molecule blending is an effective strategy for improving the mechanical properties of poly(vinyl alcohol) (PVA). However, the influence of structural differences among phenolics on the toughening mechanism remains unclear. In this study, we systematically investigated the reinforcing effects of three trihydroxy-phenolics (apigenin, galangin, and baicalein) on PVA hydrogels by combining molecular simulations with experimental validation. Molecular simulations predicted that H-bonding crosslinking between phenolics and PVA chains is the central reinforcement mechanism, and that the spatial arrangement of hydroxyl groups and the competition between intermolecular H-bonding ultimately determine the performance. Structural characterization and experimental validation showed that apigenin, owing to the dispersed spatial distribution of its three hydroxyl groups, exhibits stronger H-bonding crosslinking ability and achieves the most significant reinforcement. After a single freeze–thaw cycle, all trihydroxy-phenolics/PVA composite hydrogels exhibited markedly better mechanical properties than pure PVA hydrogels, with the following strength order: apigenin/PVA > galangin/PVA ≈ baicalein/PVA. This study provides a rapid and efficient theoretical model and a reference case for small-molecule toughened PVA hydrogels.

## 1. Introduction

Poly(vinyl alcohol) (PVA) hydrogels have broad application prospects in biomedicine, tissue engineering, and flexible electronics owing to their excellent hydrophilicity, good biocompatibility, and low toxicity [1,2,3,4]. However, PVA hydrogels prepared by conventional methods often suffer from insufficient mechanical strength and poor toughness, limiting their use in demanding environments [5]. In recent years, various strategies have been developed to improve the mechanical properties of PVA hydrogels. The introduction of nanofillers such as graphene, cellulose nanocrystals, or MXene into the PVA matrix can significantly enhance mechanical properties through interfacial interactions, but uniform dispersion of these nanofillers remains challenging [6,7,8,9]. Although dual-network entanglement or metal-ion coordination crosslinking can achieve property improvements, the introduction of foreign substances may adversely affect processability and biocompatibility [10,11,12]. Overall, existing methods either involve complex fabrication processes or provide limited enhancement of mechanical properties, and there is still a need for simpler and more efficient reinforcement strategies.

Small-molecule blending, as a green and controllable physical modification method, has gained attention in recent years [13,14,15]. Natural phenolics are rich in phenolic hydroxyl groups that can form intermolecular hydrogen bonds with the alcoholic hydroxyl groups on PVA chains, thereby constructing a multiple crosslinking network and effectively enhancing the mechanical properties of the hydrogel. For example, although supramolecular hydrogels formed by tannic acid (TA) and PVA have been extensively studied, the strong hydrogen bonding (H-bonding) between tannic acid and PVA often leads to instantaneous irreversible aggregation, making homogeneous mixing difficult even at 90 °C [16,17,18]. In contrast, small-molecule phenolics can form a more controllable H-bonding network with PVA chains. However, the structure–property relationship between phenolics and the reinforcement effect, particularly how the number and spatial arrangement of hydroxyl groups precisely affect crosslinking ability, remains poorly understood at the molecular level [19,20,21], and the screening of new reinforcing agents relies largely on empirical trial and error with low efficiency [22].

Trihydroxy-phenolics are a class of natural hydrophobic products widely present in plants, characterized by diverse structures and rich biological activities [23,24,25]. In theory, due to their lower number of hydroxyl groups and strong π–π stacking interactions, such phenolics may not form significant cross-linking effects with PVA to the same extent as tetrahydroxy- or pentahydroxy-phenolics [26,27]. Whether trihydroxy-phenolics can act as effective H-bonding bridges to enhance the network structure under such conditions is a question worth in-depth investigation.

In this study, we first employed all-atom molecular dynamics (AAMD) simulations and density functional theory (DFT) calculations to predict the binding modes, H-bonding counts, and binding energies between three trihydroxy-phenolics (apigenin, galangin, baicalein) and PVA chains, and to forecast the relative order of reinforcement effects. Subsequently, the simulation predictions were systematically validated by rheological testing and structural/morphological characterization. Experimentally, none of the three trihydroxy-phenolics could induce gelation of PVA at room temperature, but after a single freeze–thaw cycle, all composite hydrogels exhibited significantly better mechanical properties than pure PVA, and the reinforcement effect followed the order predicted by simulations: apigenin/PVA > galangin/PVA ≈ baicalein/PVA. This study not only reveals the potential of trihydroxy-phenolics as efficient reinforcing agents for PVA hydrogels, but also provides a theoretical basis and a reference example for rapidly screening natural phenolic small-molecule enhancers.

## 2. Results and Discussion

### 2.1. Molecular Structure Analysis

**Scheme 1:**
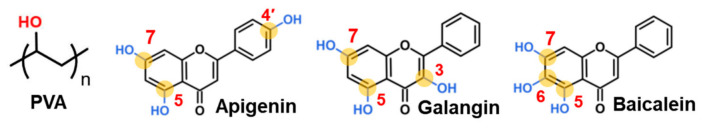
Molecular structures of PVA, apigenin, galangin, and baicalein.

As shown in Scheme 1, three bioactive natural trihydroxy-phenolics with distinct hydroxyl group distributions were selected. Apigenin (Api) has 5,7,4′-trihydroxy groups located on two different aromatic rings, spatially well dispersed. Galangin (Gal) has 5,7,3-trihydroxy groups, with the 3-OH located on an oxygen-containing heterocycle. Baicalein (Bai) has all 5,6,7-hydroxyl groups concentrated on the same aromatic ring, forming a densely arranged vicinal group. They share the same 5-OH and 7-OH groups, while the third hydroxyl group is in a different position, making them ideal for a direct comparison.

**Figure 1:**
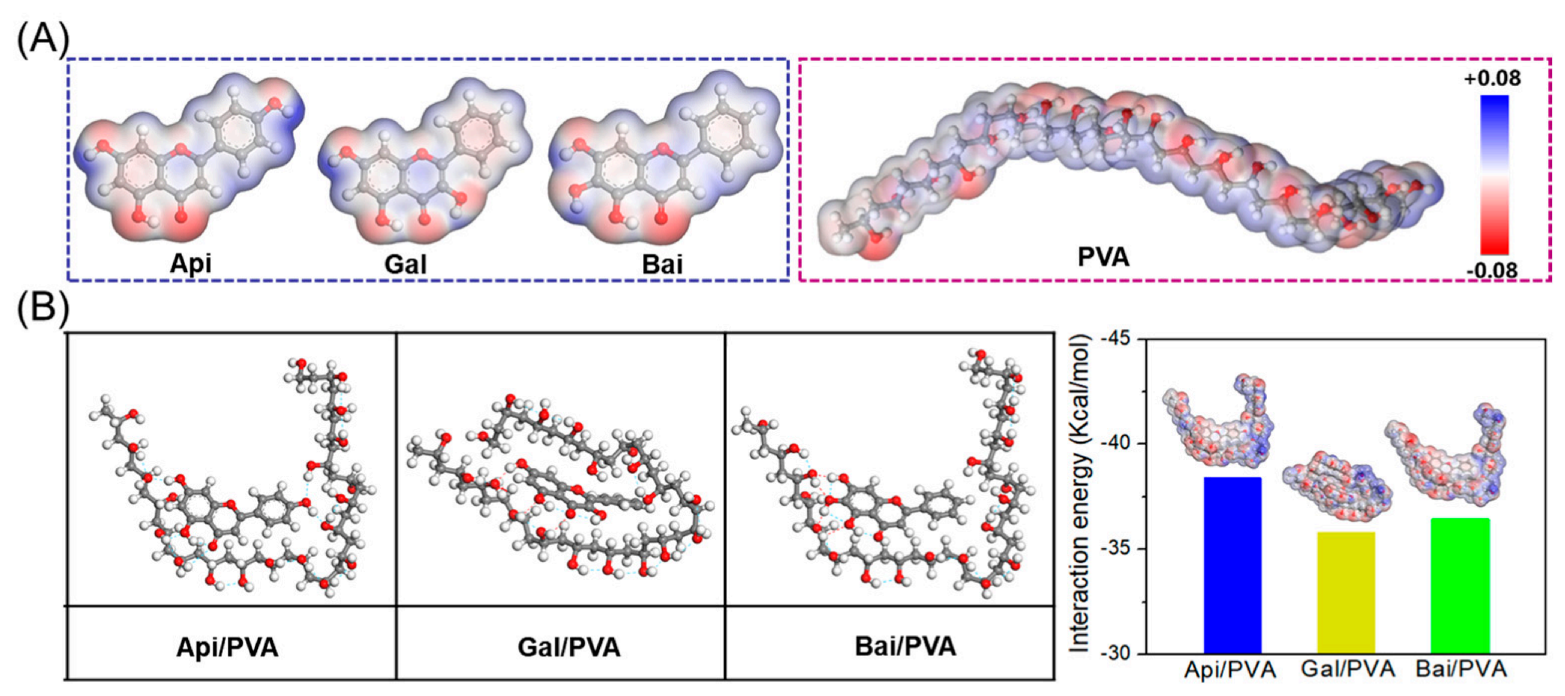
(**A**) ESP distribution maps for PVA and trihydroxy-phenolics (red: negative charge; blue: positive charge). (**B**) Interaction energies between Api, Gal, Bai, and PVA.

To predict the H-bonding interactions between the trihydroxy-phenolics and PVA, electrostatic potential (ESP) distributions were first calculated using DFT. DFT solves the electronic structure of a molecule by approximating the exchange–correlation functional, thereby providing the electron density distribution and the resulting electrostatic potential (ESP). The ESP maps visualize regions of positive and negative electrostatic potential, which directly indicate the hydrogen-bond donor (positive) and acceptor (negative) sites, and thus allow for a qualitative prediction of the preferred H-bonding patterns between the phenolics and PVA. H-bonding is essentially an electrostatic attraction between positive and negative charges on different atoms [28,29]. As shown in Figure 1A, for a single hydroxyl group, the oxygen atom carries a negative ESP value while the hydrogen atom carries a positive ESP value. Api exhibits dispersed electrostatic regions for its three hydroxyl groups; Gal shows a relatively uniform ESP distribution; and Bai, with its dense hydroxyl groups, has a concentrated ESP distribution, which favors H-bonding. Additionally, we calculated a randomly configured PVA chain with a degree of polymerization of 20, which contains multiple alcoholic hydroxyl groups. Notably, in molecules such as Gal, the 5-OH and 3-OH can form intramolecular H-bonding with adjacent carbonyl (C=O) groups, potentially affecting their binding to PVA.

To compare the binding ability of the trihydroxy-phenolics with PVA, the interaction energies (E(int)) between different hydroxyl groups and a PVA model molecule were calculated [30]. Formation of H-bonding stabilizes the system and lowers the intermolecular interaction energy. The calculation results show that Api, with its dispersed structure, has an E(int)with PVA of approximately −38.5 kcal/mol, whereas Bai, bearing vicinal trihydroxy groups, gives an E(int) of −36.8 kcal/mol (Figure 1B). Furthermore, the π–π stacking dimer structures of Api, Gal, and Bai exhibited the lowest E(int) of −39.80 kcal/mol and the highest of −35.50 kcal/mol (Figure S1). The binding strength between the trihydroxy-phenolics and PVA is not significantly higher than their self-interactions, but the possibility of forming H-bonding interactions still exists.

### 2.2. All-Atom Molecular Dynamics (AAMD) Simulations

**Figure 2:**
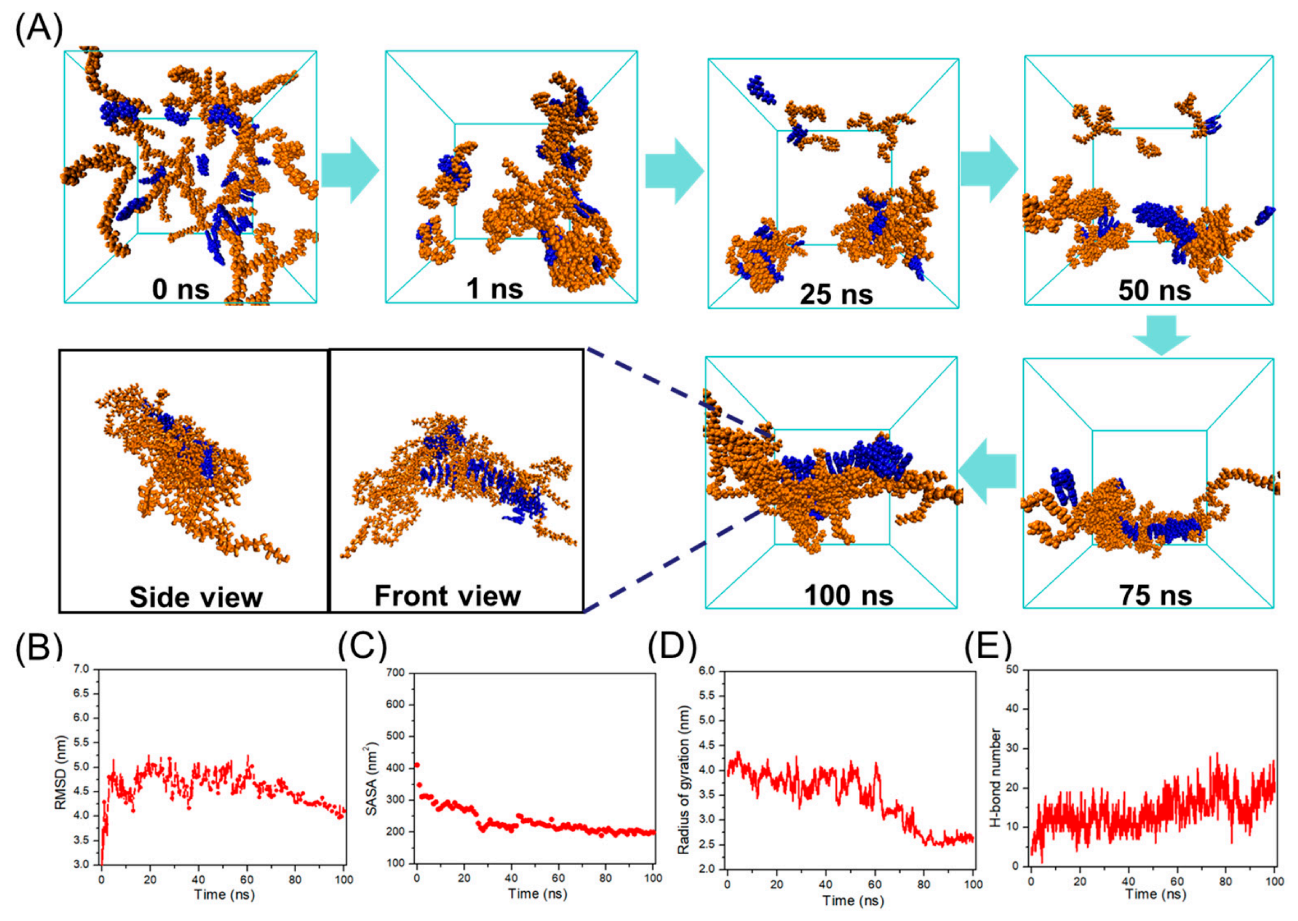
AAMD simulations of the Api/PVA system. (**A**) Structural changes in the Api/PVA system per 25 ns during the simulation. (**B**) RMSD, (**C**) SASA, (**D**) Rg, and (**E**) H-bond count of the Api/PVA system during the simulation period.

AAMD were further employed to investigate the interactions between trihydroxy-phenolics and PVA. Taking the Api/PVA system as an example, 25 Api molecules and 25 PVA chains were initially placed randomly in a cubic box filled with water, equilibrated at 298.15 K for 1 ns, and then subjected to 100 ns of MD simulation. Throughout the simulation trajectory, apigenin molecules were observed to gradually aggregate. As shown in Figure 2A, structural analysis at 25 ns intervals indicated that the morphology of the aggregates stabilized during the last 25 ns of the simulation.

Analysis of various system parameters revealed that the root-mean-square deviation (RMSD) stabilized after 20 ns (ca. 7.2 nm), indicating that the system reached equilibrium. The solvent-accessible surface area (SASA) decreased markedly within the first 30 ns, suggesting reduced solvent exposure due to intermolecular aggregation. The radius of gyration (Rg) gradually decreased during the first 70 ns, reflecting a transition from a dispersed state to a compact aggregate. The number of hydrogen bonds in the Api/PVA system stabilized at approximately 20 after 80 ns, whereas the Gal/PVA and Bai/PVA systems stabilized at about 12 and 10, respectively (Figures S2 and S3). These results indicate that aggregate formation likely arises from intermolecular H-bonding, which is significantly favorable for intermolecular association.

Importantly, the H-bonding between them is likely to further enhance the various properties of PVA. Further analysis of the H-bonding formed by each hydroxyl group in the trihydroxy-phenolics revealed that: the three hydroxyl groups of Api each independently form H-bonding with PVA chains, providing the highest total number of binding sites; the 3-OH and 5-OH of Gal participate in crosslinking but contribute less due to competition from intramolecular H-bonding; the 3-OH, 6-OH, and 5-OH of Bai are sterically crowded and suffer from spatial hindrance, resulting in the lowest overall binding efficiency (Figure S4). Therefore, the simulations clearly predict the reinforcement order as Api/PVA > Gal/PVA ≈ Bai/PVA.

### 2.3. Preparation of Trihydroxy-Phenolics/PVA Complexes

**Figure 3:**
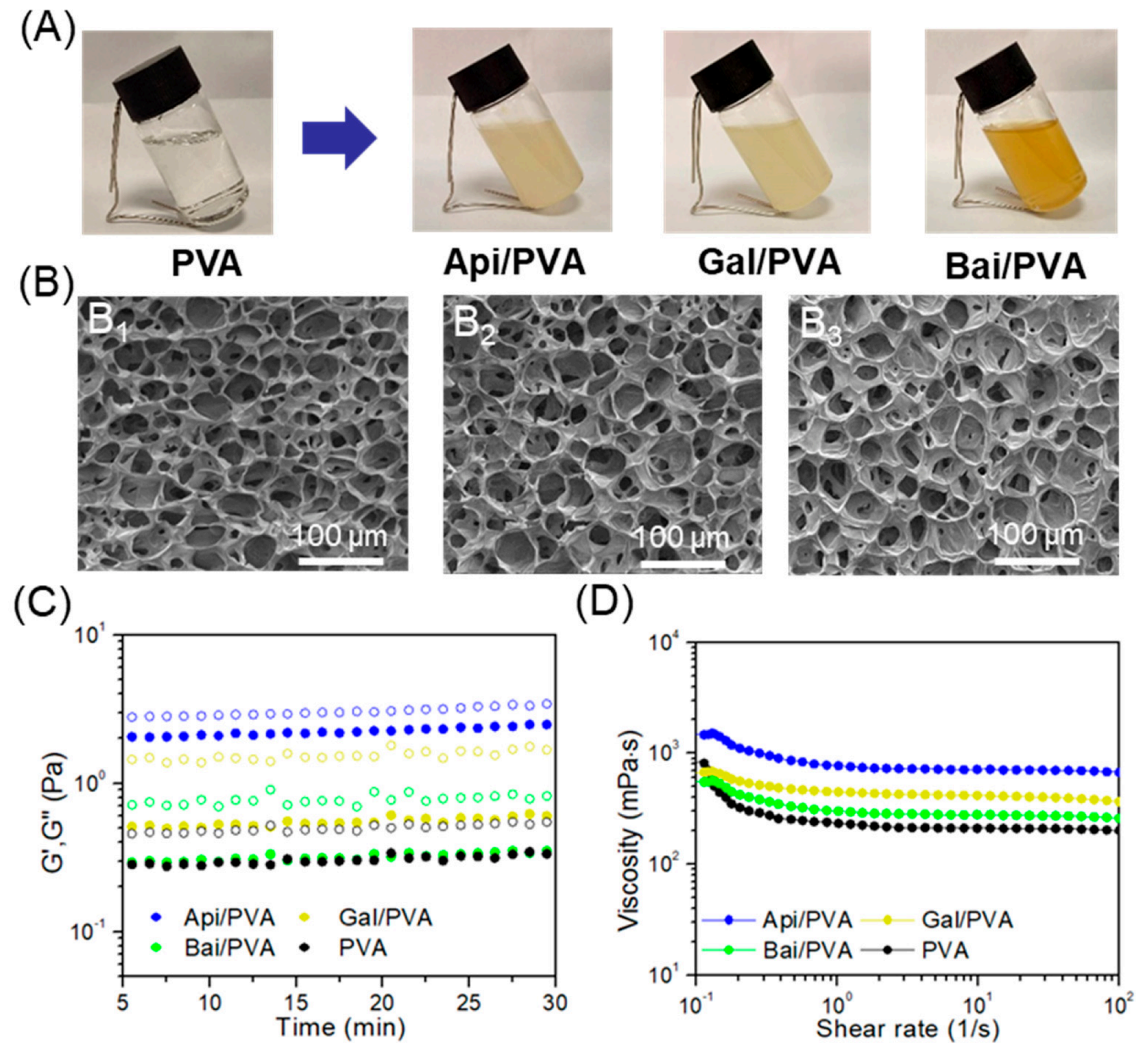
(**A**) Digital images of PVA, Api/PVA, Gal/PVA, and Bai/PVA complexes. (**B**) SEM images, (**C**) rheological testing, and (**D**) viscosity testing of trihydroxy-phenolics/PVA complexes. The storage modulus (G′) is represented by solid dots, and the loss modulus (G″) is represented by hollow dots. B1, B2, and B3 represent the SEM images of Api/PVA, Gal/PVA, and Bai/PVA complexes, respectively.

Trihydroxy-phenolics/PVA complexes were prepared by solution blending. PVA (10 wt%) was dissolved at 90 °C, and 0.06 wt% of Api, Gal, or Bai was added. The complexes were homogenized and then allowed to stand at 25 °C for 24 h. The vial inversion test confirmed that all complexes remained fluid and did not form self-supporting hydrogels (Figure 3A). The morphology of the complexes was characterized by scanning electron microscopy (SEM). As shown in Figure 3B, the freeze-dried trihydroxy-phenolics/PVA complexes exhibited irregular pore walls and loose pores, composed of fibers with diameters of 25–50 µm. The three-dimensional porous structure between fibers can provide storage space for water molecules [31]. All three freeze-dried complexes rapidly absorbed water within 25 min during swelling tests and gradually reached equilibrium after 90 min (Figure S5). To rule out the possibility that the absence of gelation was due to an inappropriate concentration, we screened phenolic loadings from 0.02 to 0.1 wt%. In all cases, the mixtures remained fluid after standing at 25 °C for 24 h, indicating that trihydroxy-phenolics alone cannot induce PVA gelation at 25 °C.

The rheological behavior of the three trihydroxy-phenolics/PVA complexes was studied using a rheometer [32,33]. When the storage modulus (G′) is less than the loss modulus (G″), the sample is in a typical solution state; otherwise, it is in a gel state. At room temperature (25 °C) and an angular frequency of 0.5 rad/s, the time sweeps showed that G″ of the three complexes was consistently nearly an order of magnitude larger than G′, indicating good fluid characteristics (Figure 3C). Moreover, the G′ value of Gal/PVA (0.5 Pa) was higher than that of Bai/PVA (0.3 Pa), but lower than that of Api/PVA (2.1 Pa). Viscosity tests at shear rates of 0.1–100 s^−1^ showed the same trend (Figure 3D), with Api/PVA exhibiting consistently higher viscosity than the other two. The moduli of the three complexes followed the predicted order: Api/PVA > Gal/PVA ≈ Bai/PVA. Notably, the viscosity of these complexes was significantly higher than that of pure PVA solution, indicating that these phenolics all have the potential to improve PVA properties. In addition, due to the clustering of its three hydroxyl groups, the cross-linking efficiency between Bai and PVA is low, resulting in a weak reinforcing effect. Combined with the MD simulation results, the non-interfering 7-OH, 5-OH, and 4′-OH groups of Api can serve as three stable H-bond binding sites, but the insufficient number may explain why the Api/PVA complex cannot gelate. It is worth noting that variations in certain parameters (e.g., chain length and additive concentration) could substantially influence the H-bonding crosslinking density and the resultant mechanical properties, and we plan to systematically investigate these factors in subsequent studies.

### 2.4. Formation Mechanism

**Figure 4:**
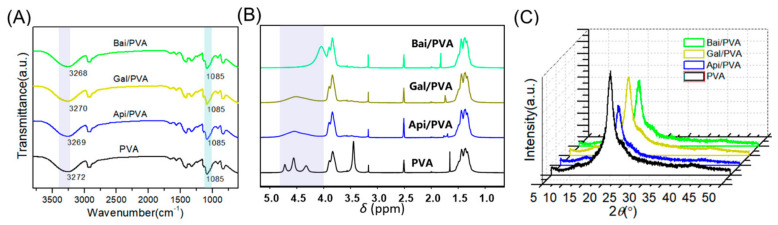
(**A**) FT-IR spectra, (**B**) ^1^H NMR spectra, and (**C**) XRD patterns of the trihydroxy-phenolics/PVA complexes. The PVA concentration was fixed at 10 wt%, and the trihydroxy-phenolics concentration was fixed at 0.06 wt%.

To further demonstrate the existence of H-bonding interactions between trihydroxy-phenolics and PVA, multiple structural characterization methods were employed [34]. As shown in Figure 4A, the O–H stretching vibration peak of pure PVA is located at approximately 3272 cm^−1^. In the trihydroxy-phenolics/PVA complexes, this peak shifted to lower wavenumbers (Api/PVA: 3269 cm^−1^; Gal/PVA: 3270 cm^−1^; Bai/PVA: 3268 cm^−1^), which is consistent with the characteristic redshift typically observed upon formation of hydrogen bonds in similar TA/PVA systems reported in the literature [35]. In the ^1^H NMR spectrum of pure Api (in DMSO-d_6_), the hydroxyl proton signals (Figure S6) were observed at δ 12.97 (5-OH), 10.82 (7-OH), and 10.02 (4′-OH), and pure PVA exhibited three hydroxyl proton peaks at δ 4.33, 4.56, and 4.72, and an HDO peak at δ 3.45. Upon addition of trihydroxy-phenolics, the hydroxyl proton peaks of PVA broadened and merged, the HDO peak intensity decreased and eventually almost disappeared, and a new broad peak appeared near δ 4.50 (Figure 4B and Figure S7). This phenomenon is attributed to the formation of H-bonding between the active hydroxyl groups, which reduces intermolecular distances and promotes hydrogen proton transfer and exchange among HDO, phenolics, and PVA.

Furthermore, the H-bonding between trihydroxy-phenolics and PVA affects the intermolecular interactions of PVA, thereby hindering the crystallization of PVA (2θ = 19.6°, 22.9°, and 40.8°) [36,37]. As shown in Figure 4C, upon introduction of trihydroxy-phenolics, the intensity of the PVA crystalline peaks decreased. The decrease was most pronounced for the Api/PVA complex, followed by Bai/PVA and then Gal/PVA, the latter being relatively weaker. The crystallinity of pure PVA calculated from the XRD patterns was 42.5%, higher than that of Bai/PVA (38.2%), Gal/PVA (36.5%), and Api/PVA (33.1%). The phenolic molecules inhibited the crystallization of PVA, and the degree of crystallinity reduction was closely related to the H-bonding strength, also following the order predicted earlier: Api/PVA > Gal/PVA ≈ Bai/PVA. Notably, the moderate crystallinity decrease is offset by the extra H-bonding crosslinks from phenolics, which collectively contribute to the enhanced mechanical performance shown below.

### 2.5. Preparation of Trihydroxy-Phenolics/PVA Composite Hydrogels

**Figure 5:**
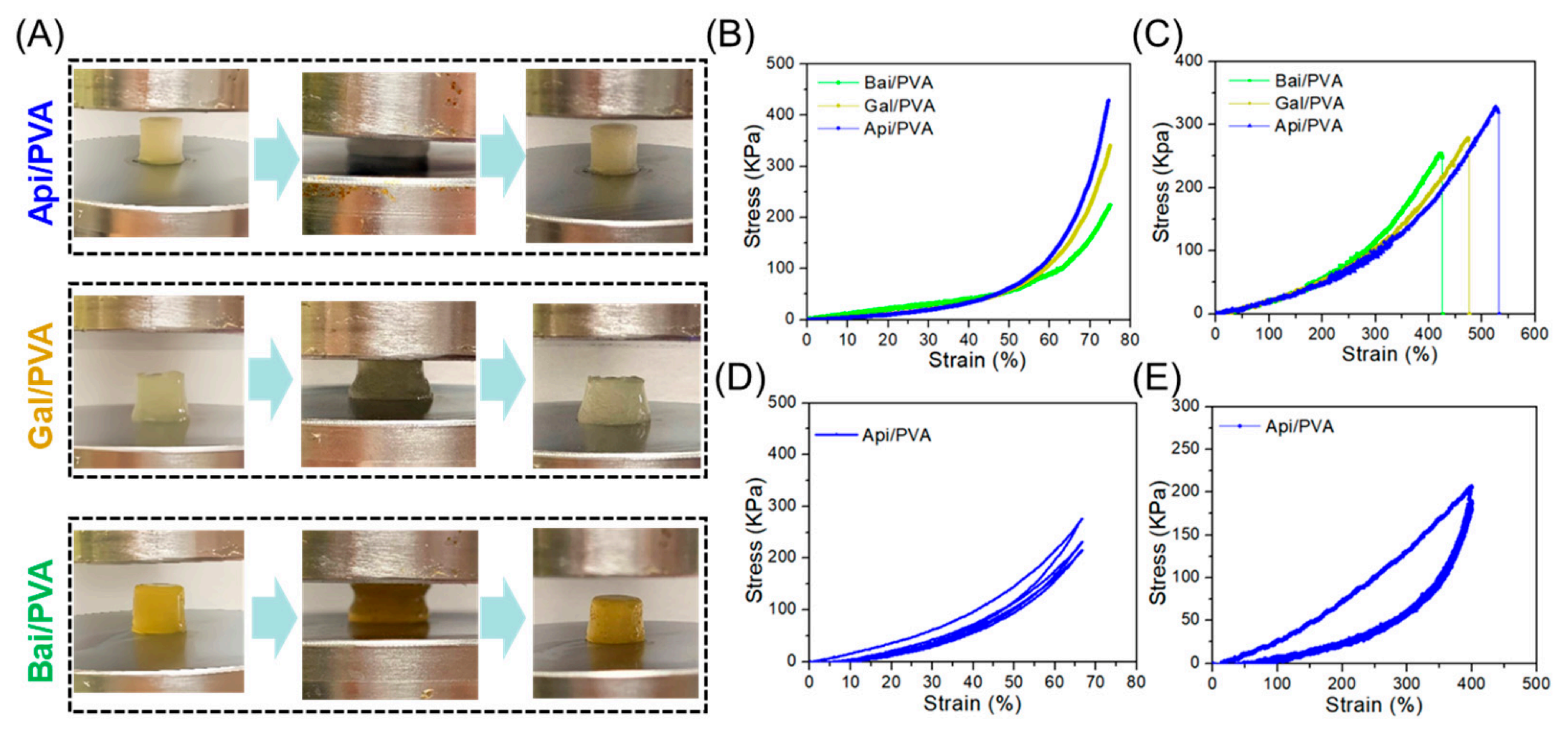
(**A**) Different effects of trihydroxy-phenolics/PVA composite hydrogels after compression (75% strain). (**B**) Compression and (**C**) tensile tests of hydrogels (PVA: 10 wt%; phenolics: 0.06 wt%). (**D**) Loading–unloading compression and (**E**) tensile tests of Api/PVA composite hydrogel.

The trihydroxy-phenolics/PVA complexes were further processed using a freeze–thaw method. The mixed solutions were injected into molds, frozen at −20 °C for 12 h, and then thawed at 25 °C for 3 h. After one cycle, all samples formed self-supporting hydrogels. Under the same conditions, pure PVA also formed hydrogels, but they were soft and easily deformed, whereas the trihydroxy-phenolics/PVA composite hydrogels exhibited significantly higher compressive strength and tensile elasticity (Figure 5A and Figure S8). These composite hydrogels exhibited good strength and toughness, with no deformation or rupture after compression or stretching. As shown in Figure 5B, compression tests were performed on the hydrogels with a fixed strain of 75%. The stress–strain curves indicated that the compressive strength of pure PVA hydrogels at this strain was only 220 kPa (Figure S9). Api/PVA composite hydrogel also showed the highest compression strength, reaching 440 ± 12 kPa, which was 1.37 times that of Gal/PVA (320 ± 8 kPa), 1.69 times that of Bai/PVA (260 ± 6 kPa), and more than twice that of pure PVA hydrogel [37,38,39]. In fact, their moduli are all significantly lower than those reported for PVA/TA composites, which may be due to the significant difference in the number of hydroxyl groups, as a single TA molecule contains more than 50 hydroxyl groups [40].

Moreover, the Api/PVA hydrogel also exhibited the best performance in tensile tests, with tensile strength and elongation at break reaching 310 kPa and 550%, respectively, which are 1.2 times and 1.5 times those of pure PVA (Figure 5C). More importantly, in loading–unloading tests at 65% strain, the hydrogel showed elastic behavior with a small hysteresis after the first cycle. The curves of the second and third cycles almost completely overlapped without significant strength decay (Figure 5D,E), indicating that it can withstand external forces to some extent. These results demonstrate that the H-bonding interactions between trihydroxy-phenolics and PVA have a significant reinforcing effect on PVA hydrogels, following the order Api/PVA > Gal/PVA ≈ Bai/PVA.

We also evaluated the effect of the trihydroxy-phenolics/PVA composite hydrogels on normal cell viability (Figure S10). Mouse fibroblast (L929) cells were incubated with the three hydrogels for different time periods, then a green fluorescent nuclear dye was added to observe the viability of live cells, and a CCK-8 assay was used to assess cell survival. The results showed that after co-culture for 24 h, 48 h, and 72 h, L929 cells in the groups containing phenolic-containing hydrogels all exhibited high viability, indicating that the prepared hydrogels have good biocompatibility [41,42]. These properties lay a foundation for the application of trihydroxy-phenolics/PVA composite hydrogels in biomedical fields such as tissue engineering and drug delivery.

## 3. Materials and Methods

### 3.1. General Information

#### 3.1.1. General Materials

The natural phenolics (apigenin, galangin, and baicalein) used in the experiments were purchased from Sigma-Aldrich (Shanghai, China). Poly(vinyl alcohol) (PVA, type 1799, degree of hydrolysis 98–99%) and other chemicals were purchased from Aladdin Reagent Co., Ltd. (Shanghai, China). All chemicals were used as received without further purification, and deionized water was used throughout the experiments.

#### 3.1.2. Preparation of Complexes

PVA powder (10 g, 10 wt%) was mechanically stirred with deionized water (90 g) at 90 °C for 30 min to obtain a homogeneous solution. Nitrogen was then bubbled into the solution for 0.5 h. Under nitrogen protection, a phenolic powder (0.06 wt% relative to the mass of PVA, i.e., 0.006 g) was added to the PVA solution, and stirring was continued while reheating at 80 °C for another 30 min. The resulting solution was then ultrasonicated using an ultrasonic cleaner (power: 100 W) at room temperature (25 °C) for 10 min to remove bubbles. Finally, all products were allowed to cool statically at room temperature (25 °C) to obtain the trihydroxy-phenolics/PVA complex solutions. For each of the three phenolics (apigenin, galangin, and baicalein), the same procedure was followed.

#### 3.1.3. Preparation of Hydrogels

Hydrogels were prepared by the classic freeze–thaw method. The complex solution described above was injected into a mold, frozen at −20 °C for 12 h, and then thawed at room temperature (25 °C) for 3 h. After the freeze–thaw treatment, gel formation was determined by the vial inversion method: a sample that did not flow upon inversion was considered a hydrogel.

### 3.2. Characterization

#### 3.2.1. Scanning Electron Microscopy (SEM)

The samples were quickly frozen in liquid nitrogen slush for 30 s and then sputter-coated with gold. Morphology was observed using a scanning electron microscope (Quanta250, FEI, Hillsboro, OR, USA) at an accelerating voltage of 5 kV.

#### 3.2.2. Fourier Transform Infrared Spectroscopy (FTIR)

PVA and phenolic/PVA samples were deposited onto KBr pellets and dried under ambient conditions. FTIR spectra were collected in the range of 400–4000 cm^−1^ at room temperature (25 °C) using an iS50 spectrometer (Thermo Nicolet, Madison, WI, USA).

#### 3.2.3. X-Ray Diffraction (XRD)

Hydrogels dried under ambient conditions were placed on a non-diffractive silicon wafer. XRD patterns were obtained using an X-ray diffractometer (D8 Advance, Bruker AXS Co., Ltd., Karlsruhe, Germany) with Cu-Kα radiation over a 2θ range of 5–60°.

#### 3.2.4. ^1^H NMR Spectroscopy

After freeze-drying, Api, PVA and phenolics/PVA samples with different ratios were dissolved in dimethyl sulfoxide-d_6_ (DMSO-d_6_). ^1^H NMR spectra were recorded on a Bruker AVANCE III spectrometer at 400 MHz.

### 3.3. Calculation Methods

#### 3.3.1. Density Functional Theory (DFT)

The initial structures of trihydroxy-phenolics and PVA molecules were constructed using Avogadro software 1.2.0 [43] and underwent a simple optimization. Based on these, structure optimization was performed using the open-source ORCA 4.2.0 software [44], and the conformation with the lowest energy was selected for subsequent calculations. Structures were optimized using the B3LYP method [45] with D3-BJ dispersion correction and the def2-SVP basis set. Electrostatic potentials of the phenolics and PVA were also calculated using this software. Subsequently, complexes of the phenolics with PVA were constructed and optimized with ORCA 4.2.0. The weak intermolecular interaction energies of the complexes were calculated using the above method with the ma-def2-SVP basis set [46], and basis set superposition error (BSSE) correction was applied [47]. The interaction energy was calculated according to: E(interaction) = E(AB) + E(BSSE) − E(A) − E(B). Results were visualized using VMD 1.9.3 software [48].

#### 3.3.2. All-Atom Molecular Dynamics (AAMD)

AAMD simulations were performed using the GROMACS (2022.6) [49] software package with the GAFF2 force field [50] and the TIP3P water model. The GAFF2 force field parameters are well developed and widely used for simulating the assembly processes of small organic molecules and simple polymers. Charges were calculated using the restrained electrostatic potential (RESP) formalism with the Multiwfn 3.7 package [51,52]. The ratio of PVA to phenolics was fixed at 1:1. Initially, 50 molecules were randomly placed in a cubic box with a side length of 60 Å. After solvation with water molecules, energy minimization was first performed using the conjugate gradient algorithm with a maximum force tolerance of 200 kJ/mol. The temperature and volume of each system were then equilibrated by running a 500 ps constant-pressure (NPT) simulation. Subsequently, a 100 ns production simulation was carried out under the NPT ensemble. Under constant temperature and pressure conditions, the system temperature was maintained at 300 K and the pressure at 1 bar. The cutoff distance for non-bonded interactions was 1.2 nm, and long-range electrostatic interactions were treated using the particle mesh Ewald (PME) method. All covalent bonds involving hydrogen atoms were constrained using the LINCS algorithm. Results were visualized using VMD 1.9.3 software.

### 3.4. Performance Research

#### 3.4.1. General Rheological Test

Rheological properties of the hydrogels were measured using a modular compact rheometer (Anton Paar, MCR 302, Graz, Austria). In the time-sweep test, the strain was fixed at 0.5%, the angular frequency at 0.5 rad/s, and the temperature at 25 °C.

#### 3.4.2. Compression Mechanical Test

For compression tests, the three types of trihydroxy-phenolics/PVA hydrogel samples were prepared as cylindrical specimens (diameter: 20 mm; thickness: 12 mm). In the compressive strength test, the maximum compressive strain was set to 75%, and the strain rate was set to 10 mm/min. At least three parallel specimens were tested for each sample, and the compressive strength value was taken as the average of multiple measurements. All mechanical tests were performed at room temperature (25 °C). Engineering stress σ = F/S, where S is the cross-sectional area of the specimen and F is the load. Engineering strain ε = (h − h_0_)/h_0_ × 100%, where h is the change in thickness and h_0_ is the initial thickness of the specimen.

#### 3.4.3. Tensile Mechanical Test

All mechanical properties of the hydrogels were measured using a biomechanical testing machine (23 MTS Insight, MTS Systems Co., Ltd., Eden Prairie, MN, USA). Dumbbell-shaped bulk hydrogels (length: 12 mm; width: 4 mm; thickness: 2 mm) were stretched with clamps at a tensile rate of 100 mm/min. Three samples were tested for each experimental point. The tensile strain corresponds to the change in tensile strength, and the elongation of the specimen is the tensile stress and strain at break. Engineering strain ε = (h − h_0_)/h_0_ × 100%, where h is the change in height and h_0_ is the initial height of the specimen.

### 3.5. Bioactivity Testing

#### Biocompatibility Test

L-929 cells were cultured in RPMI-1640 medium supplemented with 10% (*v*/*v*) fetal bovine serum and 1% penicillin–streptomycin in a humidified incubator at 37 °C with 5% CO_2_. The cells were seeded into 96-well plates at a density of 1 × 10^4^ cells/mL and incubated for 24 h. After replacing the medium with serum-free medium for 24 h, Api/PVA, Gal/PVA, and Bai/PVA hydrogels were added and incubation continued for another 24 h, 48 h, and 72 h. The old medium was then removed, and 0.1 mL of medium containing 10% (*v*/*v*) CCK-8 reagent was added to each well, followed by further incubation in the incubator (37 °C, 5% CO_2_) for 2.5 h. The absorbance at 450 nm was then measured using a microplate reader (Huisong Technology Development Co., Ltd., Shenzhen, China). L929 cells were seeded into 96-well plates at 10,000 cells/well and co-cultured with the test samples for 24 h. The cells were stained with a Calcein AM/propidium iodide (PI) cell viability kit for 30 min, and then cell viability was observed under an inverted fluorescence microscope (Leica DMI8, Wetzlar, Germany). Cell viability was calculated as: cell viability (%) = [(OD(treated) − OD(blank)/(OD(control) − OD(blank)) × 100%].

## 4. Conclusions

This study systematically revealed the reinforcing effects of trihydroxy-phenolics on freeze–thawed PVA hydrogels and their structure–property relationship by combining molecular simulation predictions with experimental validation. AAMD and DFT accurately predicted the hydrogen-bond binding abilities of the three trihydroxy-phenolics with PVA chains and the reinforcement order. Experiments confirmed that trihydroxy-phenolics cannot induce gelation of PVA at room temperature, but with the assistance of freeze–thaw processing, they become effective physical crosslinkers through intermolecular H-bonding. Structural characterization and experimental validation indicated that the reinforcement effect depends on the spatial arrangement of the three hydroxyl groups: Api, with its dispersed hydroxyl groups and absence of intramolecular competition, exhibited the strongest crosslinking ability and imparted the highest mechanical strength to the hydrogel; Bai, with densely packed hydroxyl groups prone to intramolecular H-bonding, showed the weakest reinforcement. This study not only provides a simple and green approach to high-performance PVA hydrogels but also establishes a rational design model based on molecular simulation.

## Data Availability

The original contributions presented in this study are included in the article/Supplementary Material. Further inquiries can be directed to the corresponding authors.

## Supplementary Materials

The following supporting information can be downloaded at: https://www.mdpi.com/article/10.3390/molecules31152595/s1, Figure S1. The π-π-stacking dimer structures and interaction energies of Api, Gal, and Bai; Figure S2. AAMD simulations of the Gal/PVA system. (A) Structural changes of the Gal/PVA system per 25 ns during the simulation. (B) RMSD, (C) SASA, (D) Rg, and (E) H-bond count of the Gal/PVA system during the simulation period; Figure S3. AAMD simulations of the Bai/PVA system. (A) Structural changes of the Bai/PVA system per 25 ns during the simulation. (B) RMSD, (C) SASA, (D) Rg, and (E) H-bond count of the Bai/PVA system during the simulation period; Figure S4. H-bonding numbers between trihydroxy-phenolics’s hydroxyl groups and PVA chain in (A) Api/PVA, (B) Gal/PVA, and (C) Bai/PVA systems; Figure S5. Swelling test of the trihydroxy-phenolic/PVA complexes (PVA:10 wt%, phenolics: 0.06 wt%); Figure S6. ^1^H NMR spectrum of the pure Api; Figure S7. Full ^1^H NMR spectrum of the PVA, Api/PVA, Gal/PVA, and Bai/PVA complexes; Figure S8. Different effects of trihydroxy-phenolics/PVA composite hydrogels after tensile tests; Figure S9. (A) Effects of pure PVA hydrogels after compression (75% strain). (B) Compression and (C) tensile tests of PVA hydrogels (10 wt%); Figure S10. Cytotoxicity testing of PVA, Api/PVA, Gal/PVA, and Bai/PVA hydrogels.
