# Supplementary material for: Molecular Simulation-Guided Design of H-Bonding-Reinforced Trihydroxy-Phenolics/Poly(vinyl alcohol) Composite Hydrogel
Source: Molecules. 2026 Jul 24;31(15):2595. doi: 10.3390/molecules31152595 (PMC13468053; doi:10.3390/molecules31152595)
Supplement: Supplementary file 1 [file molecules-31-02595-s001.zip › molecules-4409608-supplementary.pdf]

Supporting Figures S1–S10

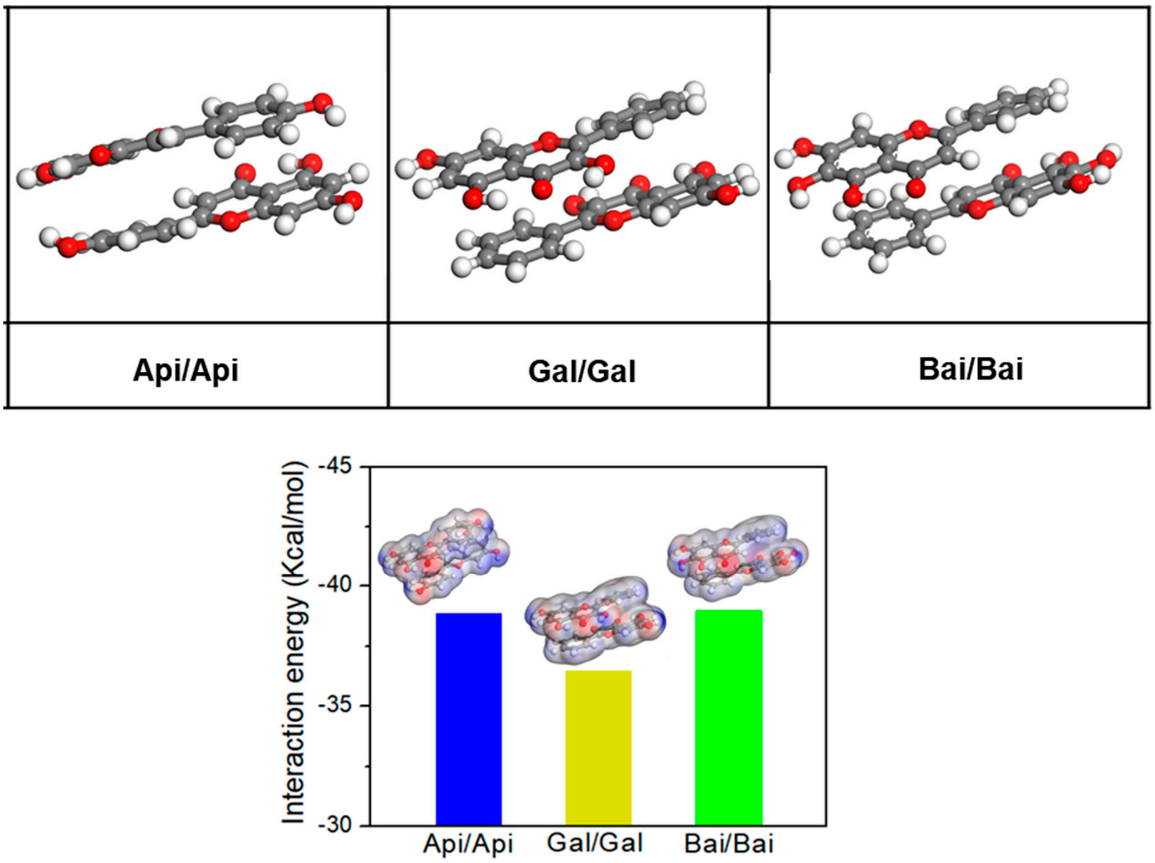

Figure. S1 The π-π-stacking dimer structures and interaction energies of Api, Gal, and Bai.

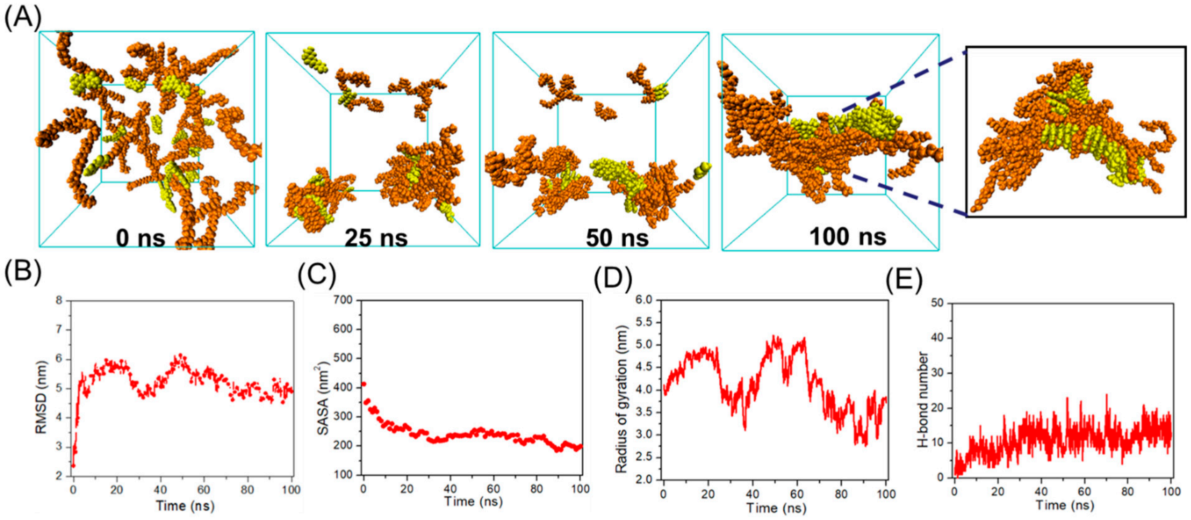

Figure S2. AAMD simulations of the Gal/PVA system. (A) Structural changes of the Gal/PVA system per 25 ns during the simulation. (B) RMSD, (C) SASA, (D) Rg, and (E) H-bond count of the Gal/PVA system during the simulation period.

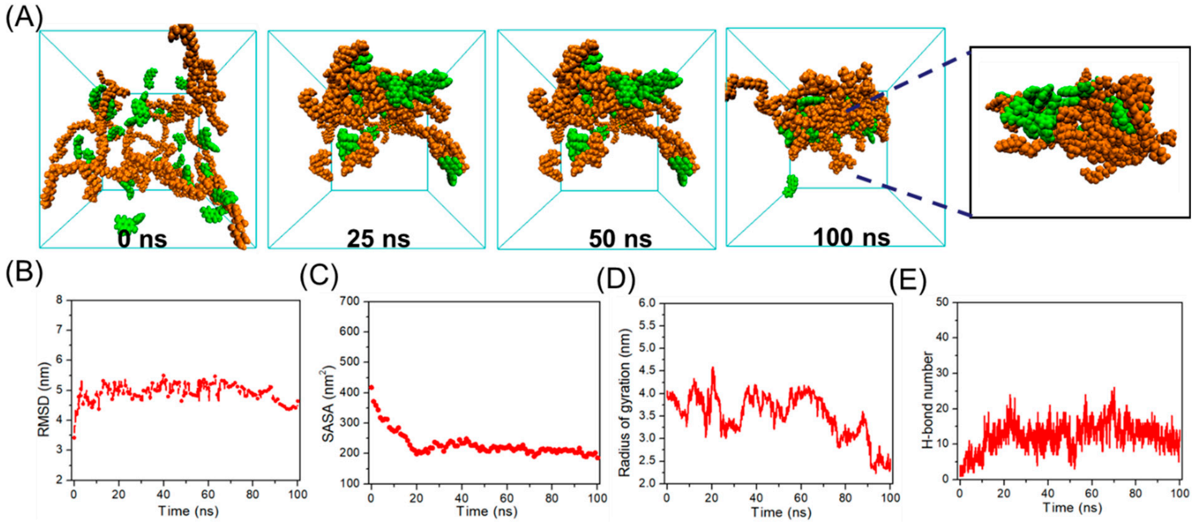

Figure S3. AAMD simulations of the Bai/PVA system. (A) Structural changes of the Bai/PVA system per 25 ns during the simulation. (B) RMSD, (C) SASA, (D) Rg, and (E) H-bond count of the Bai/PVA system during the simulation period.

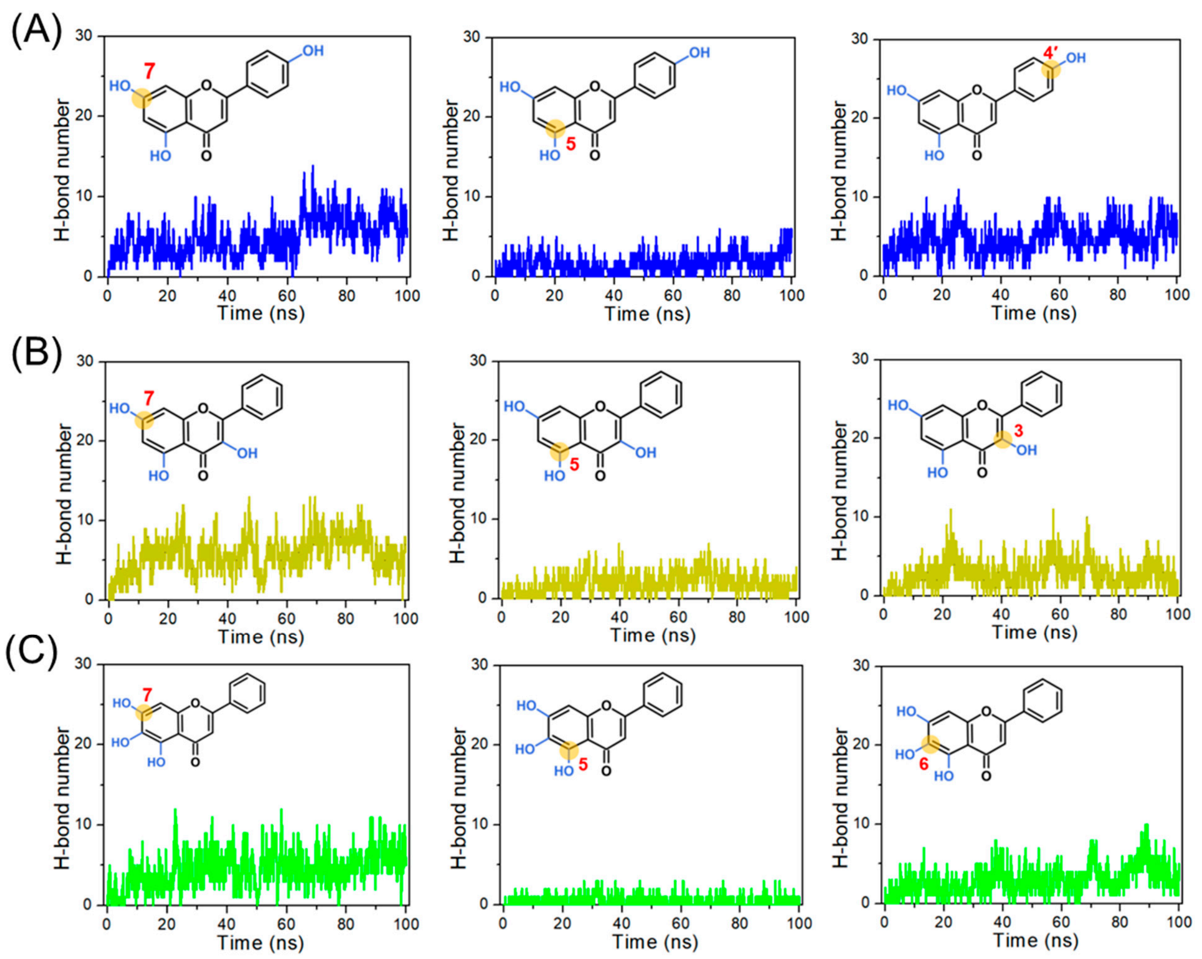

Figure S4. H-bonding numbers between trihydroxy-phenolics's hydroxyl groups and PVA chain in (A) Api/PVA, (B) Gal/PVA, and (C) Bai/PVA systems.

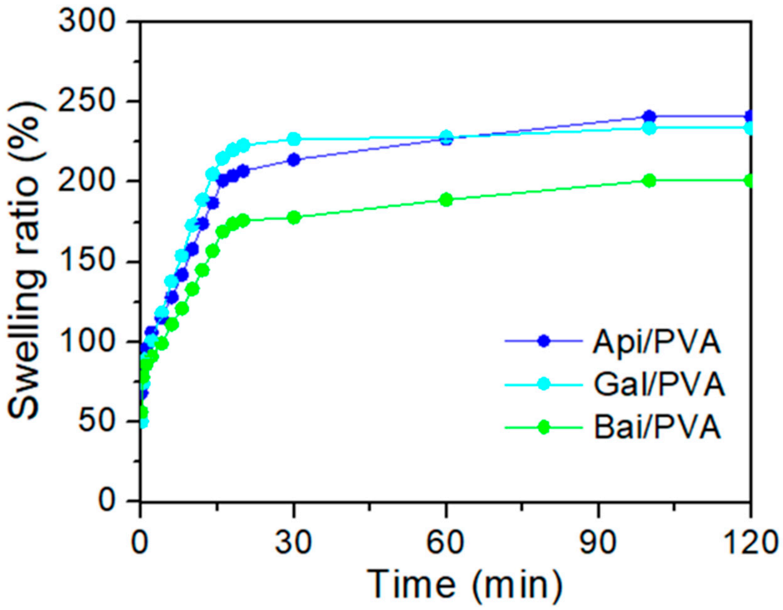

Figure. S5 Swelling test of the trihydroxy-phenolic/PVA complexes (PVA: 10wt%, phenolics: 0.06wt%).

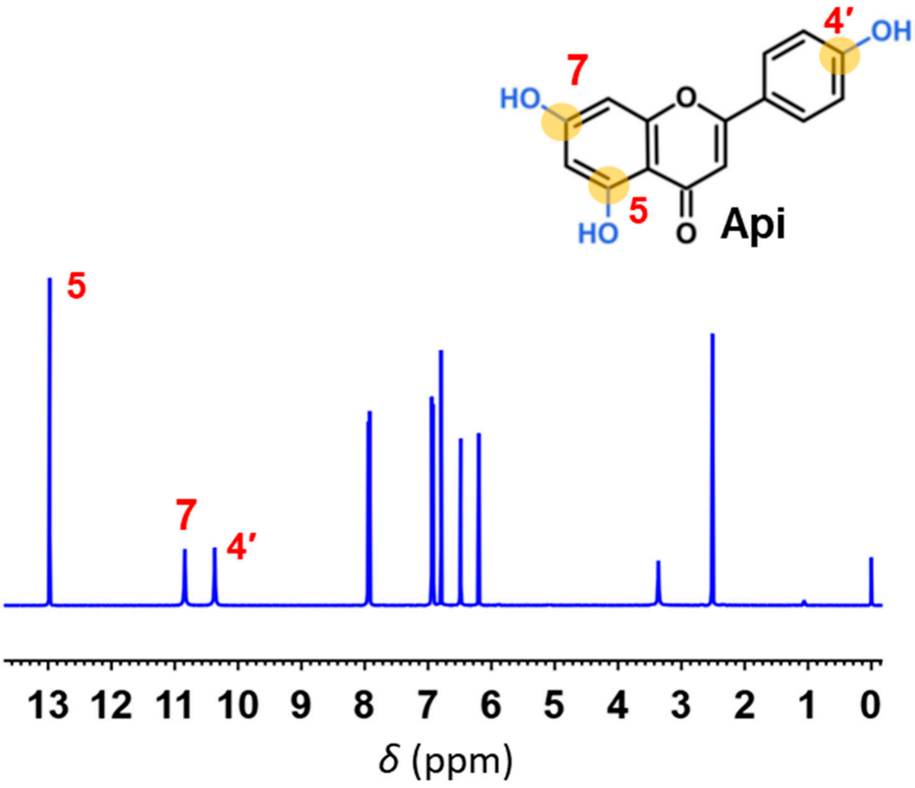

Figure. S6 ^{1}H NMR spectrum of the pure Api.

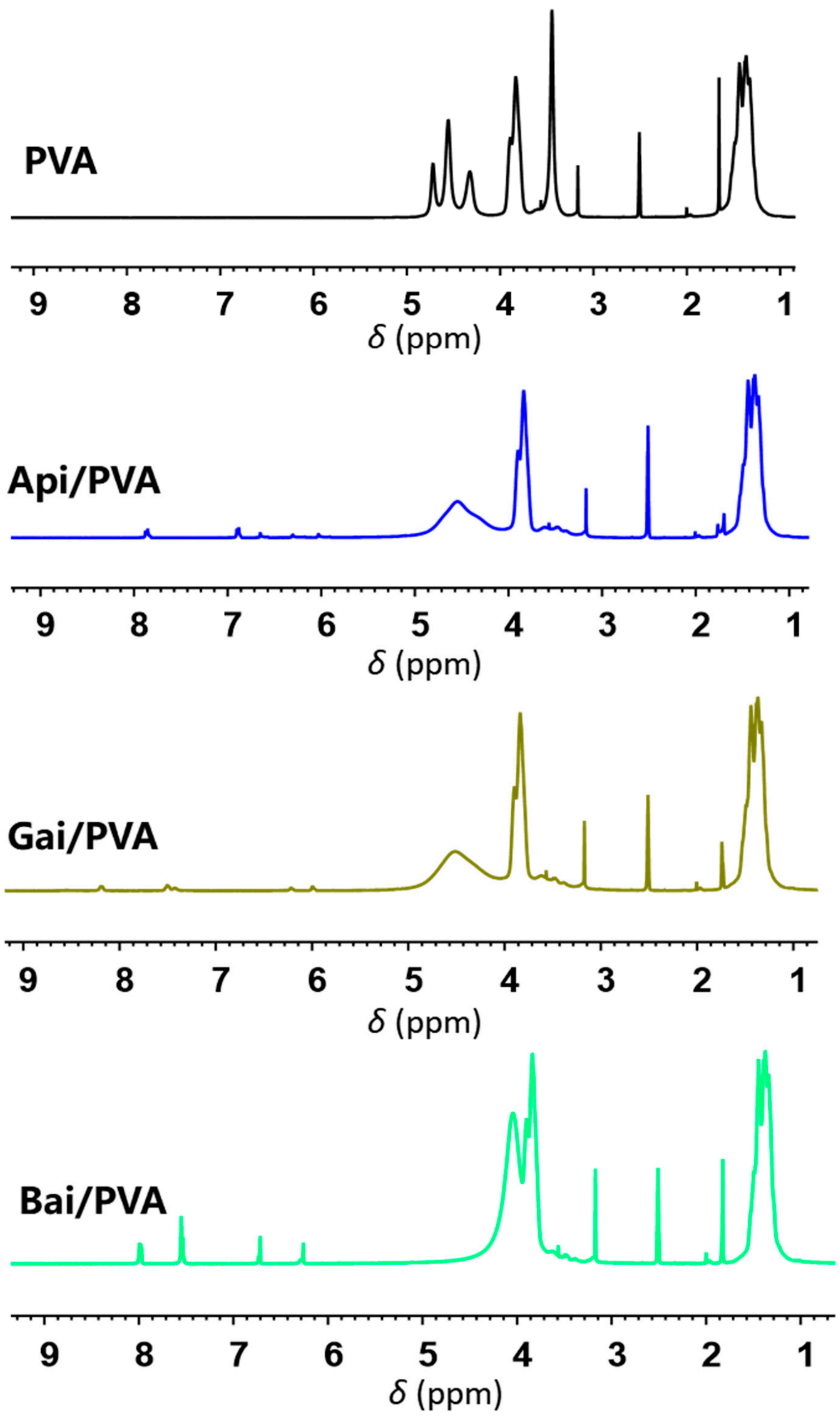

Figure. S7 Full ^{1}H NMR spectrum of the PVA, Api/PVA, Gal/PVA, and Bai/PVA complexes.

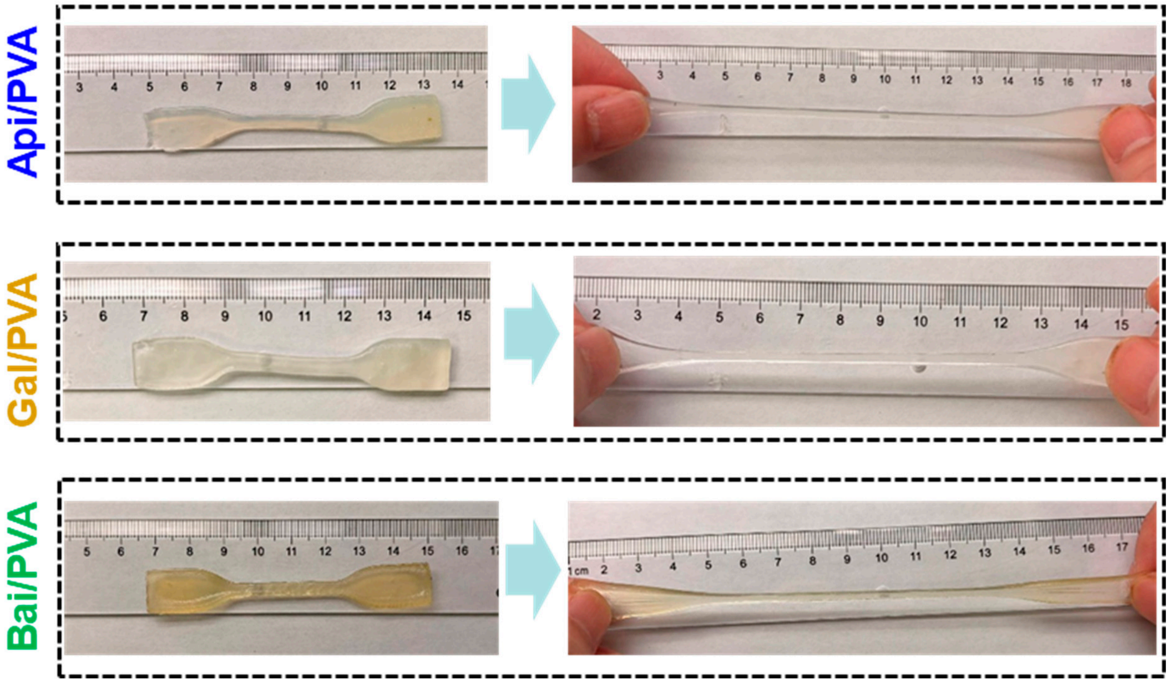

Figure. S8 Different effects of trihydroxy-phenolics/PVA composite hydrogels after tensile tests.

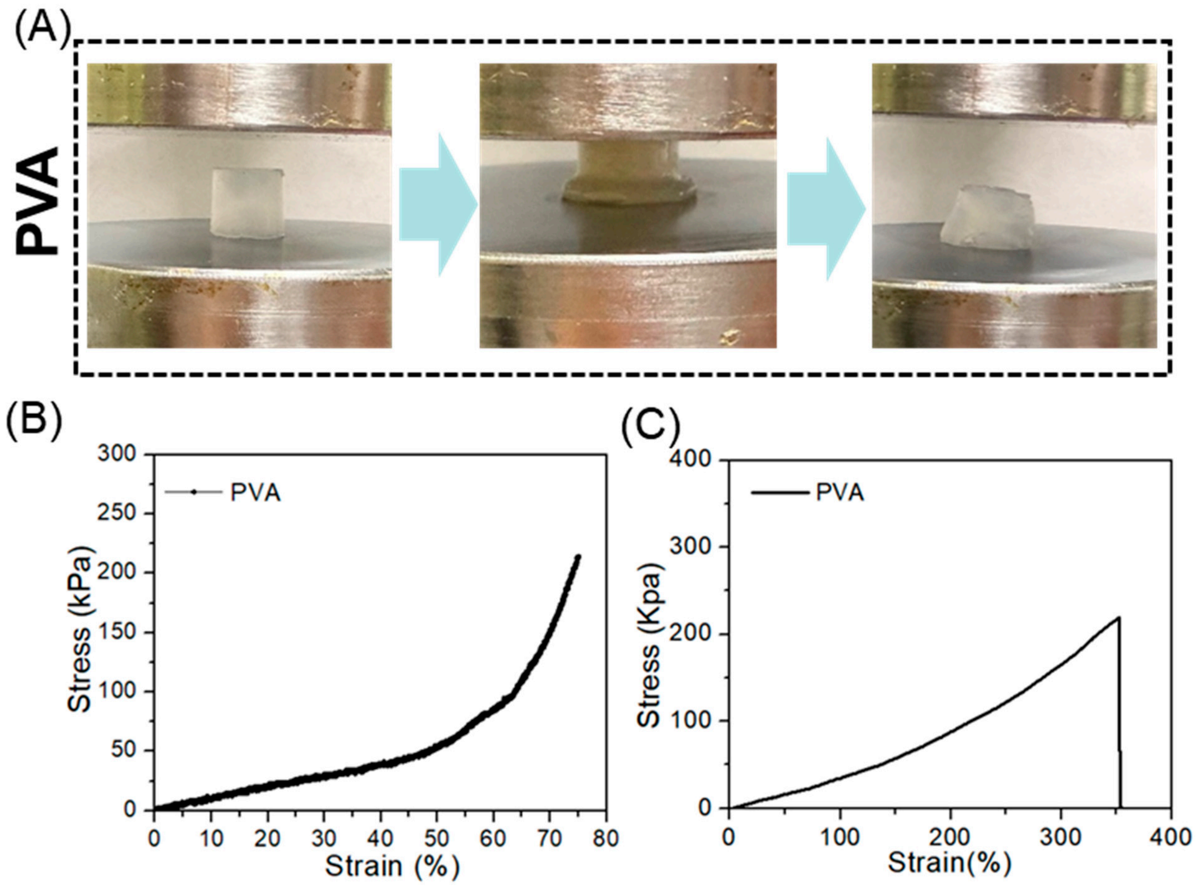

Figure. S9 (A) Effects of pure PVA hydrogels after compression (75% strain). (B) Compression and (C) tensile tests of PVA hydrogels (10 wt%).

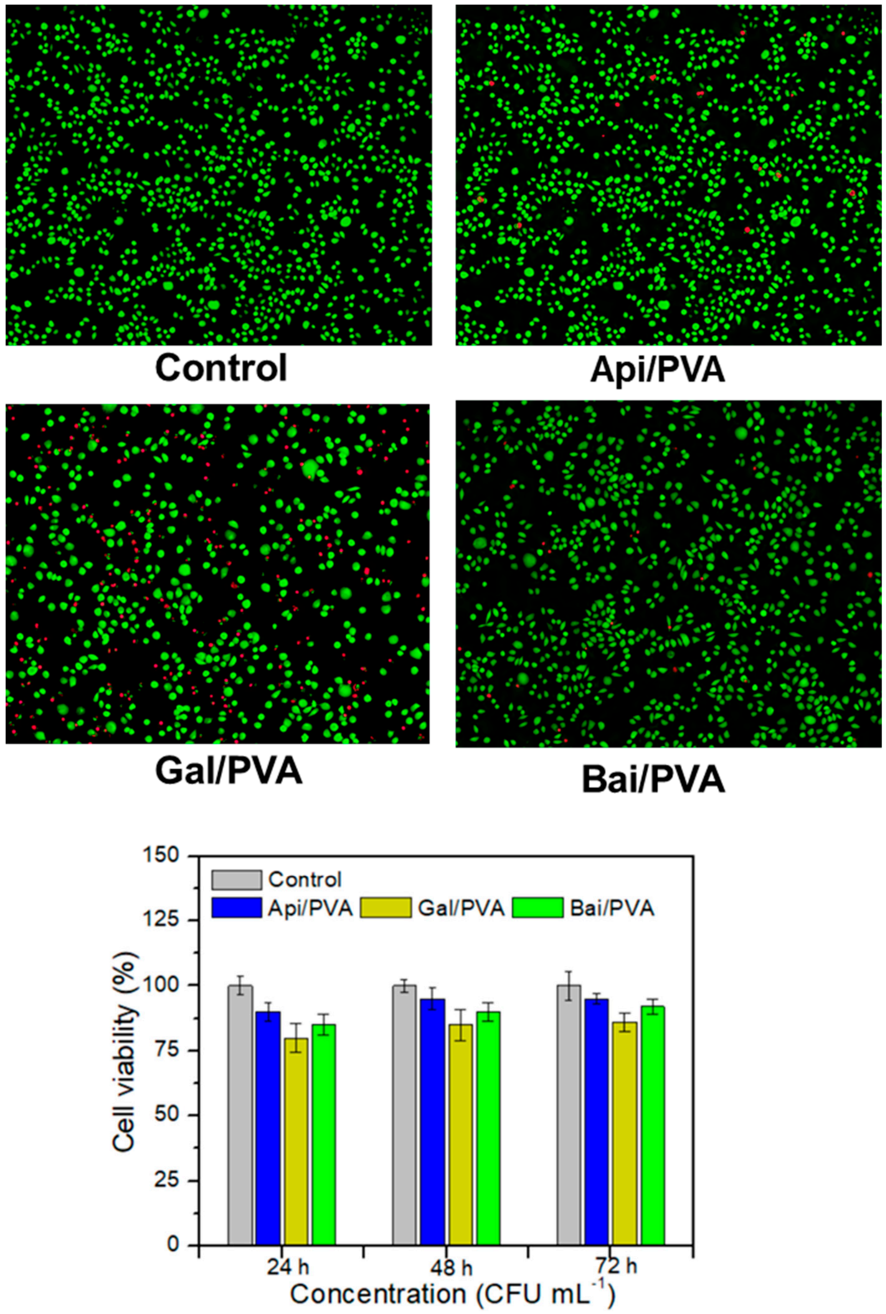

Figure. S10 Cytotoxicity testing of PVA, Api/PVA, Gal/PVA, and Bai/PVA hydrogels.
